# A Comprehensive Review on Selenium and Its Effects on Human Health and Distribution in Middle Eastern Countries

**DOI:** 10.1007/s12011-021-02716-z

**Published:** 2021-04-21

**Authors:** Marek Kieliszek, Iqra Bano, Hamed Zare

**Affiliations:** 1 Department of Food Biotechnology and Microbiology, Institute of Food Sciences, Warsaw University of Life Sciences-SGGW, Nowoursynowska 159C, 02-776 Warsaw, Poland; 2 Department of Veterinary Physiology and Biochemistry, Shaheed Benazir Bhutto University of Veterinary & Animal Sciences Sakrand, Sindh, 67210 Pakistan; 3 Cellular and Molecular Research Center, Birjand University of Medical Sciences, Birjand, Iran

**Keywords:** Selenium, Selenoproteins, Human health, Middle East, GPx

## Abstract

Selenium (Se) is an important microelement with numerous positive effects on human health and diseases. It is important to specify that the status and consumption of Se are for a specific community as the levels of Se are extremely unpredictable between different populations and regions. Our existing paper was based on the impacts of Se on human health and disease along with data on the Se levels in Middle Eastern countries. Overall, the findings of this comprehensive review show that the consumption and levels of Se are inadequate in Middle Eastern nations. Such findings, together with the growing awareness of the importance of Se to general health, require further work primarily on creating an acceptable range of blood Se concentration or other measures to determine optimal Se consumption and, consequently, to guarantee adequate Se supplementation in populations at high risk of low Se intake.

## Introduction

**Fig. 1:**
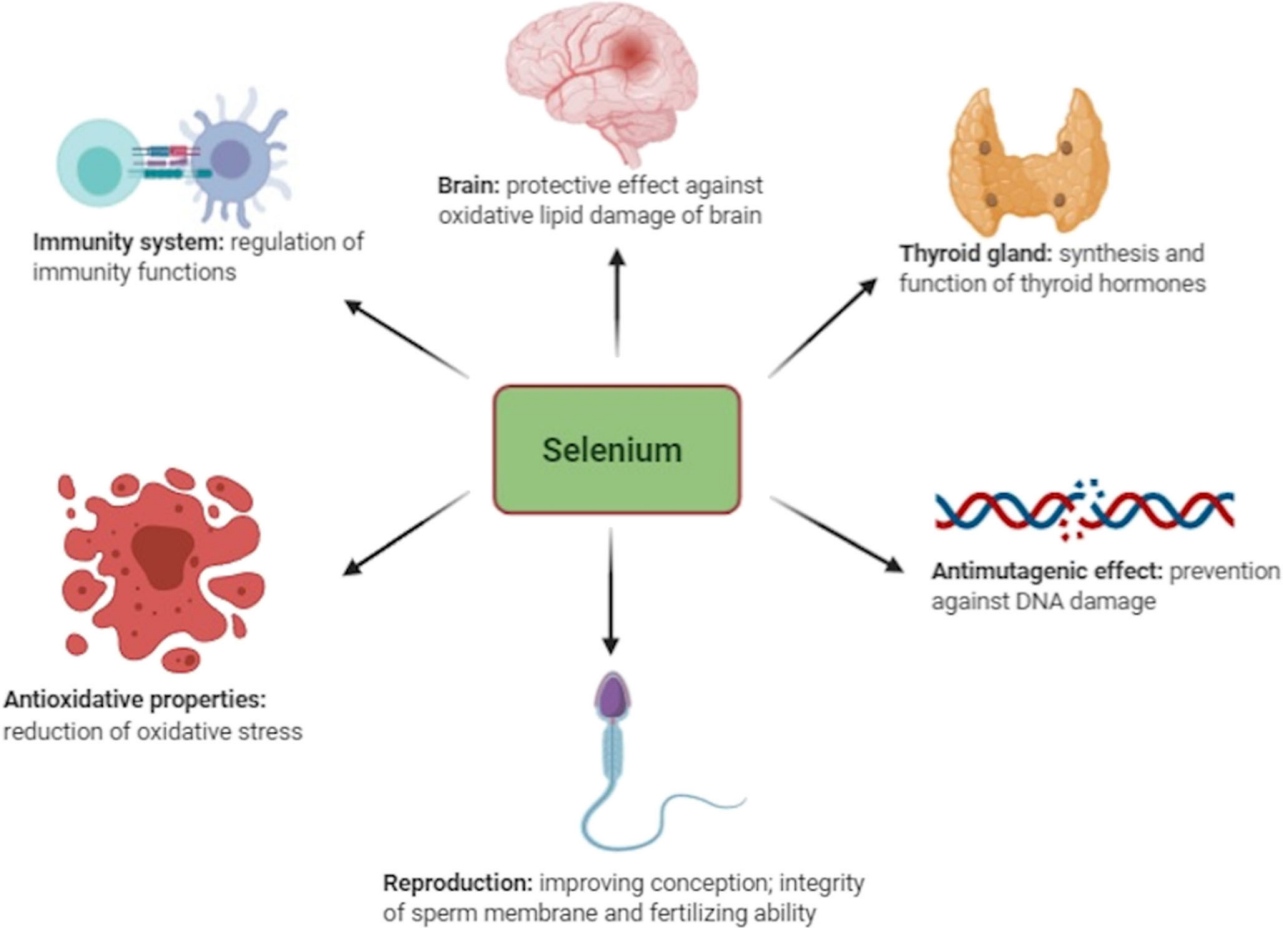
Functions of selenium in various organisms

Human body cells require appropriate nutrition for the maintenance of homeostasis. Micronutrients, such as trace minerals, antioxidants, and vitamins, are crucial for performing various regenerative processes, managing oxidative stress, and developing immunity against pathogens [1]. Selenium (Se) is one of the trace minerals that is required to maintain various functions of the body (Fig. 1). It was first discovered by Swedish scientist Jöns Jakob Berzelius in 1818 during the production of sulphuric acid [2]. Initially, it was considered to be toxic to humans, until its vital function was evidenced in the last decade [3]. Se plays an important role in preserving many natural body functions; therefore, interest in Se research has grown in many areas for public health improvement over the past few decades. The biological function of Se is related to its incorporation through selenocysteine (SeCys) into the structure of proteins important for metabolism [4]. Naturally, Se is present in both organic and inorganic forms. The organic form is the Se contained in amino acids termed as selenomethionine (SeMet) and SeCys, while the inorganic forms include selenate, selenite, selenide, and elemental Se, respectively [5]. The inorganic form of Se is a major source of dietary Se in the human population. Animals who are raised on Se-containing soil are a rich source of selenoproteins (SePs). To distribute Se across a whole country, animals should be raised in fields where there is a proper concentration of Se in soil [6]. Due to the large differences in Se detection methodologies among different laboratories, it is difficult to compare Se levels in different countries [7]. It is very important to identify the status and intake of Se in a particular population as the levels of Se are highly variable between diverse communities and regions. Throughout Middle Eastern countries, very little has been known about the level of Se intake [8]. Available research shows that the level of Se is highly heterogeneous, with some states deficient, and others oversufficient. The current review is based on the biological functions of Se and selenoproteins, the health benefits of Se, and diseases concerned with deficiency of this trace mineral along with its distribution in Middle Eastern countries including Saudi Arabia, Jordan, Turkey, Libya, Egypt, Iran, and Qatar.

## Selenoproteins (SePs)

**Fig. 2:**
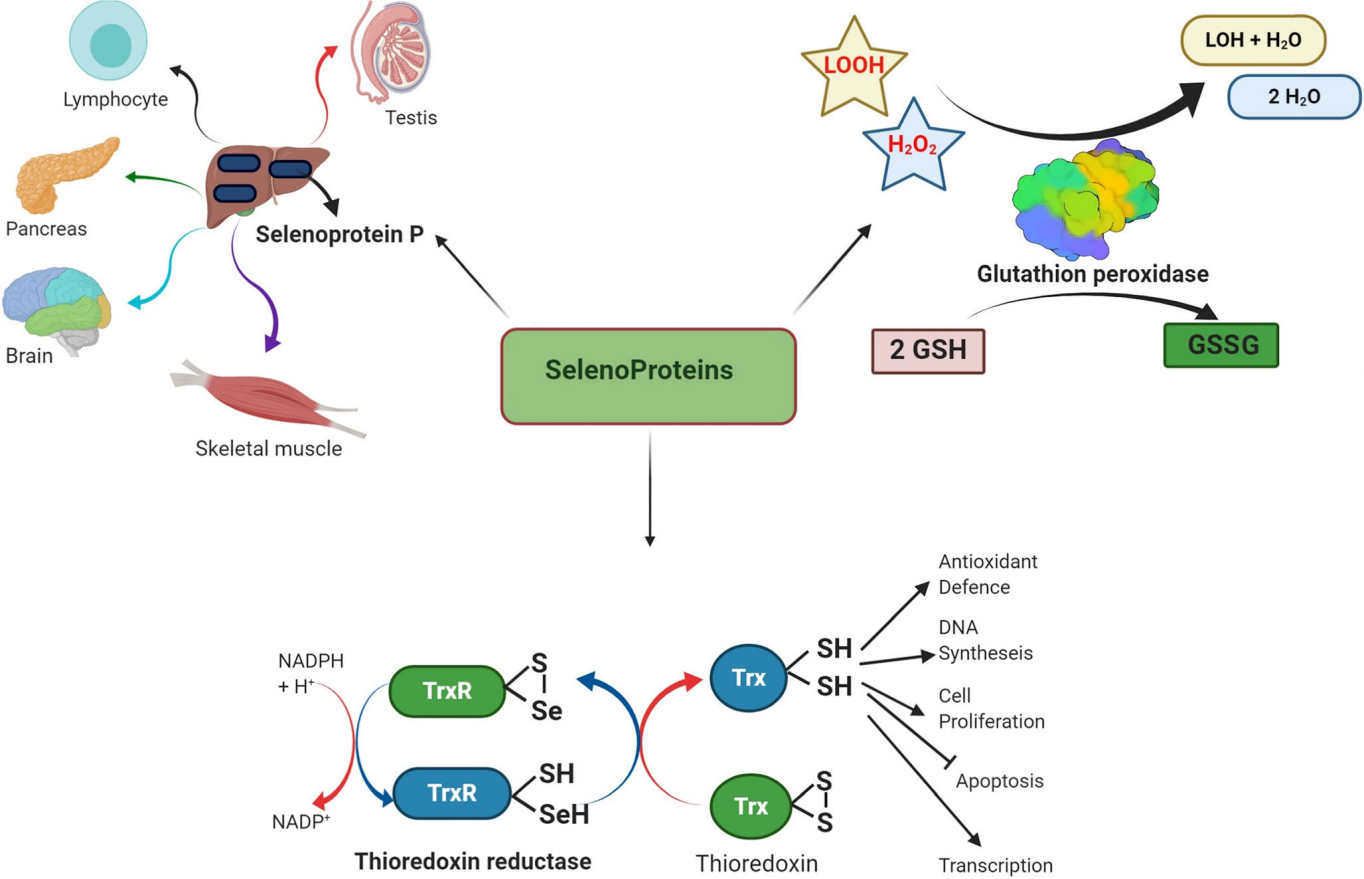
Examples of selenoproteins and their functions in organisms

SePs are considered to be proteins and perform critical biological roles [9] (Fig. 2). They were discovered by a Dr William Hoekstra of the University of Wisconsin in 1973 [10]. To date, 30 mammalian SePs have been identified from 25 genes. Structurally, they are composed of 21 amino acids [11]. During the initial phases of the analysis of SePs, recognition of SePs was mainly dependent on experimental techniques that included protein studies for Se through spectrometry and radioactivity analysis [12]. The largest group of selenoproteins is involved in the processes of protecting cells against the effects of reactive oxygen species (ROS). Generally, SePs perform their activities within cells, acting as enzymes such as deiodinases and thioredoxin, and also as antioxidants including glutathione peroxidase (GPx), which is regarded as the first animal SePs to be discovered [10]. SeP synthesis is crucial for Se maintenance and disorders occurring due to disturbance of homeostasis [13]. Both SeCys and SeMet are crucial forms of SePs and are present naturally in various sources. SeCys is found in animal tissues and in Se-containing proteins, whereas SeMet is obtained from yeast, algae, bacteria, and plants respectively [5]. Details about some SePs are given below.

## Selenoprotein-P (SelP)

SelP is a glycoprotein that was discovered in 1993 and is one of the most abundant SePs, representing some 50% of the Se in the blood. Research has shown that SelP plays a major role in selenium metabolism [5]. The reduction of systemic SelP expression is associated with Se deficiency in the diet. The consequence of these processes is the formation of oxidative stress and the occurrence of various disease states. Thus, Se deficiency reduces the content of SelP [14]. It was found that after administration of Se, the concentration of SelP increases first, and only then does the concentration of other Se-dependent proteins increase [15]. SelP is produced in hepatocytes [13]. The activity of this protein has been noted in various tissues of the body, including in plasma. It is composed of two domains: the larger N-terminal, which is responsible for maintaining the redox potential in the cell, and the smaller C-terminal, which mediates Se transport [16]. This protein is highly expressed in the testes, the brain, and liver tissues. It is considered to be a marker of Se concentration in the body. It plays a major role in the transport of Se within tissues and maintains homeostasis. SelP is the first protein discovered to contain more than one Se atom in the form of SeCys. It has also been shown that P-selenoprotein protects lipoproteins against oxidation by removing the peroxynitrite molecule that is formed due to oxidative stress within cells as a result of the reaction between superoxide ions with nitric oxide molecules at sites of inflammation [17–19]. The assumption that SelP is secreted into the bloodstream, as well as several SeCys sequences that are found in its structure, indicates that SePs can become a source of Se for peripheral tissues [20, 21]. Moreover, when a SelP-knockout mouse was fed a normal Se diet, it was marked by a substantial reduction in Se levels, particularly in the central nervous system and testes, while Se content was only slightly affected in the kidneys and other organs and tissues. In the liver, Se was also improved by removing the SelP gene and by increasing urinary excretion of Se residues [22].

## Selenoprotein W (SepW)

SepW is one of the most abundant SePs in mammals. This small 9-kDa SeP is found in the cytosol and has a high muscle and brain concentration [23]. Generally, SepW is required for muscle metabolism in animals. However, the role of this protein is still unknown. It has been revealed by some studies that its concentration was increased in animal tissues when Se was increased in the diet [24]. Moreover, skeletal muscle calcification during white muscle disease was also prevented through Se supplementation in cattle and sheep [25]. Various studies on the relationship between SepW and human muscle disease have been carried out and some researchers continue to working on this to observe SepW’s further effects on human muscle metabolism [26]. Some studies have revealed that SepW is a part of the stress-related community of SePs as its expression is heavily influenced by dietary availability of Se [27]. Another study has revealed that when excised from rat skin, a glutathione complex was found in native SepW [28].

## Thioredoxin Reductase

Thioredoxin comprises three main enzymes, including TR1, TR2, and TR3. They all act as intracellular antioxidant molecules by regulating redox reactions. These also act as a major factor for apoptosis and DNA synthesis [29]. Thioredoxin acts as a key enzyme for the metabolism of Se compounds by providing selenide for the synthesis of SePs [30]. Moreover, this SeP is known to activate the tumor suppressor p53 gene, and TR1 is highly expressed in many carcinoma cells and tumors [31]. Furthermore, the inhibition of TR1 in cancerous cell lines contributed to diminishing the proliferation of cell and cancer progression by administering knockdown TR1 cells into mice as compared to a control cell line [32]. TR1 can therefore have two conflicting functions in the development and progression of cancer. TR1 can aid cancer prevention by preserving cell redox balance and lowering the incidence of mutations that trigger the development of tumors. TR1 is also essential for tumor progression because cancer cells are highly sensitive to oxidative damage [33]. TR1 has recently been involved in mediating the essential role of Se in the immune system and prevention of HIV infection, as well as its involvement in cancer [34].

## Glutathione Peroxidase (GPx)

Glutathione peroxidase (GPx) group SePs are common in all three domains of life (archaea, bacteria, and eukarya) [35]. The bacteria, protozoa, fungi, and terrestrial plants also contain SeCys-containing GPx sequence homology. GPx carries out a variety of biological roles in cells, including the regulation of hydrogen peroxide (H_2_O_2_), hydroperoxide detoxification, and the maintenance of cell redox homeostasis [36]. GPx1 is present throughout the body but highly expressed in red blood cells, kidneys, lungs, and liver [37]. It works as an antioxidant enzyme and is thought to be affected by Se deficiency [38]. GPx2 is present in the gastrointestinal tract and protects against oxidative stress [39]. GPx3 is present in plasma and the extracellular fluid of the body and contributes to 10–30% of the Se present in the blood. It is also present in the breast, kidneys, lungs, liver, thyroid glands, heart, testes, and also in the placenta. It reduces the production of lipid hydroperoxides in plasma [40]. GPx4 is widely spread in the body but strongly expressed in the testes. It resides in mitochondria, cytosol, and the nucleus of cells. It is essential for sperm maturation and motility and has a highly effective impact on male fertility improvement [41]. GPx5 is mainly present in the olfactory epithelium and embryo cells [30]. The functions of GPx6, 7, and 8 are still thought to be unknown [42].

## Se and the Environment

Se is a very common element on Earth and is present in the lithosphere, atmosphere, hydrosphere, and biosphere respectively [43]. It is released into the atmosphere via volcanic gases. An important factor is also the emission of this element to the atmosphere from industrial sources (burning coal, crude oil) [44]. The content of Se in the atmosphere depends primarily on anthropogenic activity. Human activity releases the element into the atmosphere, making up 37.5–40.6% of total atmospheric emissions. In addition, the content of this element in the atmosphere in urban areas ranges from 1 to 10 ng/m^3^ and mainly comes from the combustion of coal and crude oil [45]. Se is unevenly distributed and concentrated in the Earth’s crust. This element is found primarily in parent rocks, sedimentary rocks of volcanic origin, and also enters to the soil together with rainfall resulting from the evaporation of water from the seas and oceans. Worldwide, its content in soil is about 0.4 mg/kg [44, 46]. Soils with a high concentration of this element are found in: North America, Ireland, Australia, and Israel. In turn, the areas poor in Se include, among others, some provinces of China (72% of the areas in China), New Zealand, and a large part of Europe [47]. Moreover, elevated Se concentrations appear in soils rich in iron compounds and organic matter in saline soils. The geochemical properties of this element will be present in the given area. Selenides and selenium sulphides occur mainly in acidic, oil-rich soils with a high content of organic matter. On the other hand, in soils with medium oxidation conditions and neutral pH, selenites (IV) predominate, which are easily soluble after combination with alkali metals. The environment is dominated by combinations of oxides with iron hydroxides (iron oxide adsorbed and fixed Se(IV)), which show low bioavailability in the soil. The third group are selenates (VI), which are found in well-oxidized alkaline soils. Selenates (VI) cannot be combined with iron compounds; therefore, they are easily available. It should be noted that individual forms of Se are easily transformed under the influence of changes in the soil environment, and inorganic forms of Se may undergo the process of biomethylation to volatile forms of Se: an example is dimethylselenide: (CH_3_)_2_Se. The distribution of individual compounds allows the natural geochemical cycle of this element to be preserved [48]. The constant cycle of Se in the environment relies on the biomethylation of elements by microbes and decomposition of the organic material present in it. As a result, volatile Se compounds are synthesized that include dimethyl selenium (DMSe), dimethyldiselenide (DMDSe), selenium oxide (SeO_2_), and hydrogen selenide (H_2_Se). The Se is present in the form of selenates and selenites in water [49]. In aqueous solutions, Se occurs most often in the form of selenite (SeO_2_^−3^) Se (IV), selenate (SeO_2_^−4^) Se (VI), biselenin (HSeO^−3^), and selenic acid (H_2_SeO_3_) [50]. The concentration of Se is elevated in groundwater as compared to seawater. The Se concentration in Poznań (Poland) is approximately 0.17–0.44 μg/L [51]. According to Stefaniak et al. [52], 8% of 3,000 samples of tap water showed an Se content exceeding the standards of the Environmental Protection Agency. The concentration of Se in polluted water may vary between 400–700 μg/L [53]. The content of Se in water depends on the geochemical environment and environmental pollution, as well as the leaching of this element from the rocks. The acceptable threshold of Se in drinking water is 10 μg/L according to World Health Organization (WHO) guidelines and the Office of Environmental Health Hazard Assessment (OEHHA) [46]. It is worth noting that Se present in soils is absorbed by plants. The absorption of this element by plants is conditioned by its chemical forms and concentration in soils. Some of these plants are identified to be present in China, which contains approximately 20.000 ppm Se concentration [54]. More than 20 seleniferous plants have been identified, one of them being the *Astragalus* species [55]. *Astragalus* includes *A. bisulcatus*, *A. racemosus*, *A. praelongus*, *A. pectinatus*, and *A. thephorosides* [54]. According to researchers, the concentration of Se is higher in green grasses than in leguminous plants [56, 57]. Plants take up Se in the form of selenite or selenate. Selenate is less absorbed by soil minerals and more easily absorbed by plants than selenite. Moreover, selenite, selenate, and selenium nanoparticles (SeNPs) in plant cells are reduced to organic compounds such as selenium amino acids (selenocysteine (SeCys)) [58]. The main consumers of Se from plants are herbivores, which are then consumed by humans. The excess or deficiency of this element at this stage of the food chain may affect the health of humans and animals. That is why it is important to monitor supplementation of this element [59].

## The Dietary Se and Human Requirements

Food enrichment with Se must be conducted in a careful and regulated manner to avoid serious impacts that are the inverse of the expected effect, since Se is among the most toxic elements in relatively small amounts, while at the very same being a vital nutrient with a significant biological role. The difference between the required quantity of Se and the toxic dose is very small [4, 60, 61]. Other sources of Se for humans are dairy products, cereals, rice, and tuna (Table 1).

**Table 1:** Selenium content in different human feeds

Feeds	Se content (mg/kg)
Beef	0.042–0.142
Lamb	0.033–0.260
Chicken	0.081–0.142
Pork	0.032–0.198
Dairy products	<0.001–0.11
Cereal	0.01–0.31
Pasta	0.01–0.10
Rice	0.05–0.08
Potatoes	0.12
Tuna	5.6
Cod	1.5
Onions	<0.5
Lentils	0.24–0.36

The level of Se within human tissue depends on dietary intake, which it related to Se availability in the soil and its geographical distribution [62]. One of the most critical problems in analytical epidemiological studies based on Se is the difficulty of measuring Se uptake from the diet [63]. According to some studies, the marker for adequate Se intake is the measurement of GPx enzyme activity, which was measured in erythrocytes, because the activity of this enzyme is directly proportional to dietary intake of Se [64]. The other methods for determining the concentration of Se include analysis of biological samples including urine, hair, and fingernails. The enteral formulas and parenteral nutrition formulas for dietary Se intake have been published since 1980 by the food and nutrition board of the US National Research Council. For parenteral solutions, sodium selenate is the best choice to be considered. The recommended amount of Se in parenteral solutions for adults is 50–70 μg/day (Table 2), while for neonates and children younger than 5 years it is 2–3 μg/day/kg weight, and for adolescents and older children the recommended dose of Se is 30–40 μg/day [65].

**Table 2:** Recommended dietary allowances (RDAs) for selenium intake [66, 67]

Group	Selenium content (μg/day)
Infants	10–15
Children 1–3	15–20
Children 4–8	20–30
Children 9–13	40–45
Adults (men, women)	55–70
Pregnant women	60
Lactating women	60–75

Nowadays, in several countries, a revolutionary technological process focused on the processing of Se-rich food items including eggs, meat, and dairy has been successfully implemented [68]. Pork and chicken enriched with Se are vailable in Korea, whereas eggs fortified with Se are now on the market of 25 countries across the globe. When reviewing research studies on the development of functional foods, it also seems clear that eggs enriched with Se could be used to supplement micronutrient deficiencies and to sustain the body’s metabolic stability [69]. An egg or chicken meat with a Se content of can supply 50% of the recommended daily intake for this ingredient [70]. Many research centers around the world are actively working to acquire food items that are sources of Se. The most attractive attribute is to have the maximum bioactivity of organic shapes of Se in food items. Moreover, the use of organic Se supplementation of yeast origin in terms of shortage has multiple beneficial effects on health [71].

## Se and Human Health

Se plays a very important role in the maintenance of various physiological processes in the human body [72]. It also plays a crucial role in the pathogenesis and pathophysiology of various disorders due to its antioxidant properties, such as oxidative stress, inflammation, apoptosis, reproductive disorders, diabetes, thyroid issues, cancer, and immune responses [8]. Rising Se deficiency in various parts of the world is leading to many pathological disorders [73]. According to the literature, it has been proved that people consuming a special diet (due to phenylketonuria) are especially vulnerable to the adverse effects of Se deficiency [74]. Patients with liver cirrhosis, celiac disease, rheumatoid arthritis, and other degenerative diseases have very low levels of Se [74]. Furthermore, individuals exposed to advanced chemotherapy, and patients who have already undergone radiation therapy, are susceptible to reduced expression of this microelement in the system [75]. Se deficiency leads to many other diseases, such as asthma due to impaired GPx activity; it also promotes impaired circulation, irregular heartbeats, coma, or sudden infant death syndrome [5, 6]. Keshan disease is another condition that is associated with Se deficiency. This is pediatric cardiomyopathy that occurs mainly in young women of reproductive age and children around 2–10 years old [76]. While excess dietary Se commonly causes food poisoning complications such as diarrhea, nausea, and vomiting [77, 78]. Many cases of excessive Se in people and livestock present in a particular geographic region are usually affected by large concentrations of Se in the soils. Animal grazing in fields in which the Se concentration is greater than 5 μg/g must be considered hazardous to consumer health. The Se derivatives are characterized by various levels of toxicity. Inorganic forms of Se have greater efficacy compared to organic versions [79]. These findings all suggest that both excessive and insufficient levels of this element have various impacts on the health of human. A range of diseases and conditions associated with Se intake or deficiency and involvement in pathogenesis are described below.

## Se and Inflammatory Response

The effect of Se on inflammatory responses was first identified in diabetic rats [80]. According to some studies, it has been proved that Se can influence inflammatory responses by inducing inhibition of NF-kB series, which promotes the production of tumor necrosis factors and interleukins [81]. Some studies suggest that there is a sex-specific impact of Se on inflammatory responses, as there is an degree of sexual dimorphism among the distribution of Se in organs of the body that affects Se status and arrangement related to body function [82]. Chronic inflammatory syndromes are usually linked with a reduction in Se status and some studies have revealed that patients with cystic fibrosis [39, 83], inflammatory bowel diesease, and acne [84] may have a lower status of Se than healthy controls. SelS (selenocysteine-containing proteins), whose secretions from liver cells and human serum recognition have also been published, is one of the SePs involved in immune responses [85, 86]. The correct supply of Se influences the process of transcription of the gene responsible for the synthesis of immunoglobulins (Ig), which plays a significant role in regulating the activity of the immune system. The Se level also influences the differentiation of macrophages. In conditions of Se deficiency, more pro-inflammatory macrophages M1 are formed, while in conditions of Se excess, mainly antiinflammatory macrophages M2 are formed. This model of action allows the induction of a pro-inflammatory cellular immune response against viral and bacterial pathogens by Th1 lymphocytes [71]. It is also worth emphasizing that the release of various mediators of inflammatory reactions in the body may trigger the occurrence of various disease states. Lowering the activity of selenoproteins may be associated with abnormalities in the functioning of the entire antiradical system in the body. SelS is regulated by inflammatory markers and extracellular sugar levels in liver cells [87]. Moreover, SelS has an antitumorigenic function in the systemic macrophages and brain astrocytes, decreasing stress [88].

## Se and Cancer

The prevention of cancer by the use of Se in humans, as well as in animals, has been indicated by various epidemiological studies. A recent study conducted on 1312 cancer patients supplemented with almost 200 μg Se on a daily basis showed a decrease in tumor size in prostate cancer, lung cancer, and colon cancer of up to 63%, 58%, and 46% respectively [89, 90]. The mechanism of action is still unknown but is thought to be through two key Se-dependent redox systems (glutathione peroxidase and thioredoxin) within cells. The specific cells that facilitate the extermination of toxin compounds and mutated cells in blood circulation are T-lymphocytes, and it is thought that Se is required for activation of T-lymphocytes [91]. It has been proved by some studies that Sec insertion sequences (SECIS: selenocysteine insertion sequence) of the open reading frame for mammalian SePs resemble that of the CD4 gene [92]. Such reading frames can encode amino acid sequences within potential selenoproteins. It is worth noting that the accumulation of UGA codons in open reading frames for selenoproteins may refer to the existence of specific sequences within the untranslated mRNA region responsible for the insertion of selenocysteine (SeCys) in place of another cysteine amino acid (Cys) during the biosynthesis of various proteins. In this way, the resulting proteins containing Se in their structure show biological activity [93]. Hence, Se is important for T-cell protein formation and its normal function [94]. Moreover, thioredoxin reductase purified from human T-cells plays an important role in reducing tumor cell growth [95]. The effects of Se status on prostate cancer are superior for secondary infections and diseases, suggesting an inhibitory effect on the spread of tumor [94]. The process of angiogenesis requires growth factors such as vascular endothelial cells, growth factors, and metalloproteinases for the degradation of proteins residing in the extracellular matrix. The metalloproteinases cause a reduction in the level of growth factors and proteins via methyl selenol (CH_3_SeH) precursor [95], whereas the selenite suppresses the invasion of human fibroids by reducing expression of metalloproteinases-2 and metalloproteinases-9 [96]. Moreover, bladder cancer development is affected by the genotype of GPx1 which suggests that GPx1 is relevant to bladder cancer progression [97]. Various methods from numerous areas including China and the USA have verified that low Se consumption is related to the occurrence of lung, thyroid, prostate, mammary and colorectal carcinomas [98]. The association between Se and risk of gastric or esophageal cancer is still unclear. Meanwhile, human experiments have been conducted with Se doses of around 50 mg/day along with a blend of additional supplements [99, 100]. To date, various compounds of Se have been tested to induce apoptosis involving various signaling pathways [101]. The results revealed that Se supplementation via organic Se decreased metastasis both in vivo and in vitro, suggesting that Se acts as an antimetastatic compound by suppressing angiogenesis, cancer cell migration, and invasion [102].

## Se and Cardiovascular Diseases

It is believed that an antioxidant plays an important role in defending against atherosclerotic and cardiovascular disease (CVD) events [103]. Deprivation of Se was strongly correlated with cardiomyopathy occurring in countries with significantly reduced Se intake [104]. Multiple studies have been conducted to evaluate Se’s effect on CVD risk [105–107]. Both small and large amounts of Se have been found to severely impact the cardiovascular system. A trial was undertaken in Eastern USA, with a 7.6-year follow-up to evaluate the impact of Se intake on CVD prevention. The occurrence of myocardial infarction, complete cerebrovascular injuries, and CVD were evaluated, and the findings showed that there is no overall advantage of applying 200 μg/day of Se to avoid CVD [108]. In general, in the context of cardiovascular physiology, the GPx family belongs to the strongest known SePs. Studies that concentrated on the significance of Se deficiency in heart disease development lacking infectious origin have already shown that correlation between an inadequate intake of Se and cardiac dysfunctions may arise from oxidative stress, as well as its complications. Animal experiments utilizing various medications and formulas of Se, as well as trials in GPx mouse models, reported an important role in neutralizing reactive oxygen and nitrogen molecules, ultimately restricting organ injury following myocardial reperfusion. In addition to the GPx subtypes, thioredoxin reductase is assumed to have specific duties in the cardiovascular system by oxidizing intraspecific and extracellular signaling molecules via an influence on adaptive responses such as remodeling [109, 110]. Moreover, some experiments based on animal models have revealed that Se deficiency has proved the down gradation of lowdensity lipoprotein (LDL) receptor, which is essential for regulating plasma cholesterol levels [111].

## Se and Alzheimer’s Disease

Alzheimer’s disease (AD) is a common type of dementia and subsequent development of disease characterized by a progressive loss of memory, expression, and recognition of individuals or objects, along with more managerial, or alert behavioral changes [112]. For many years, there has been increased interest in the role of Se in medical and neurological disorders such as AD. Furthermore, it was stated in a some animal studies that changes in the level of Se-containing compounds affect the metabolic activity of neurotransmitters [113]. This seems very significant in the study of AD because several neurotransmitters are defective in this disease. However, the most important role of Se in AD is the antioxidant role of distinct seleno-enzymes. Furthermore, the links between Se and AD risk factors have also been noted. Interactions between Se and apolipoprotein E (ApoE) and presenilin 2 are both considered to be heritable genetic factors for AD [114]. Many postmortem studies involving human brain autopsy and tissue biopsies from AD patients and control subjects have explored whether AD is related to altered levels of Se [115]. Epidemiological and clinical studies on Se have indicated stratified consequences in primary and secondary prevention of AD. Considered collectively, multiple recent studies have suggested that modification of Se is a cause of the pathogenesis of AD. However, the differences in GPx activity may not be unique to AD, because no differences in GPx activity were found when comparing the cerebrospinal fluid of patient subjects with cerebrovascular disease [116–118]. As another example, SelM (selenoprotein M), which is a SeP has been confirmed to lead to a decreased calcium stream in neurons. SelM downregulation contributed to a spike in cytosolic calcium levels [119]. This is notable because shifts in calcium homeostasis are claimed to be involved in AD pathogenesis [120, 121]. Clinically, it is important to evaluate the consequences of Se supplementation in the long term and in comparison with other possible effects to build the clinical evidence that enables an effective understanding of the impact of Se on AD prevention [112].

## Se and Type 2 Diabetes Mellitus

Several in vitro and in vivo experiments have already shown that Se plays a key role in glucose homeostasis regulation. Se has also been found to postpone diabetes development and progression. It has also been reported that Se acts as mimetic insulin in the form of selenite [122–124]. Some experiments have associated concentrations of plasma Se above approximately 140 ng/mL with enhanced type 2 diabetes (T2D) risk [125]. These results were based on the use of T2D-consistent treatment unconfirmed with glucose, insulin, or hemoglobin (HbA1c) circulation tests. However, this has not yet been verified in other randomized Se controlled studies [126]. Some animal model experiments have provided positive results. The link between a high level of Se exposure and insulin resistance and glucose intolerance in late gestation and offspring was found by contrasts between supranutritional (>190 mg Se/kg) and nutritional (<50 mg Se/kg) dietary supplementation [127]. A similar experiment with pigs has been found to show a higher circulating level insulin with a diet containing 3 mg Se/kg with natural glucose relative to dietary levels [128]. The consequences of T2D can be identified briefly as ineffective deposition and/or insulin sensitivity. SePs are essential physiological antioxidants capable of exercising insulin-like properties that may excessively impede the signaling of insulin [88]. Moreover, pancreatic beta cells demonstrate the biological plausibility of Se playing a role in T2D [129].

## Se and Thyroid Gland

Although the thyroid gland requires iodine for its normal functions, recent studies have suggested that Se status in the diet affects the volume of the thyroid gland [130]. The first ever study on Se and thyroid metabolism was carried out in central Africa with Se deficient and Se-combined diet in addition to iodine therapy. The results suggested that Se supplementation leads to a reduction in thyroid-stimulating hormone (TSH) concentration in healthy children [131]. Another study revealed that the concentration of Se was recuced in Graves’ orbitopathy disease as compared with patients having only Graves’ disease [132]. This suggests that Se deficiency may form an independent risk factor for Graves’ orbitopathy. Moreover, in another study, there was no significant link found between Se status and severity of Graves’ orbitopathy [133]. Another study suggests that Se deficiency elevates risks for hyperthyroidism, either from nodular goiter or from Graves’ disease. The alteration in thyroid hormone levels was only observed in males, signifying the sexual dimorphism concerning Se status and thyroid abnormalities [134]. Significantly, new innovative trials in mutually acute infectious thyroiditis (AIT) and Graves’ illness have the potential to change existing scientific practice where Se is an important element [135]. The determination of Se status throughout experiments and self-medication of patients is very important for clarification of outcomes. Clear recommendations for appropriate use of Se as an international guideline are urgently needed [136].

## Se and Fertility

Se is a micronutrient that is important for male and female reproduction [137]. The primary factor in male fertility maintenance is oxidative damage to sperm cells, while Se protects sperm cells from this destruction and as a result acts as a key factor in the maintenance of male fertility [102]. Low Se concentration may increase sperm susceptibility to free radicals, which may disturb the biochemical processes taking place in the acrosome [138]. It was found that lowering the Se concentration reduces sperm motility, results in damage to the central part of the sperm whip, and also increases abnormalities related to the morphological structure (mainly with an abnormal sperm head or folded tail) [139]. A prominent occurrence of free radicals and lipid peroxidation has been observed in male mice fed Se-lacking and improved Se-supplemented diets [140, 141]. The results proved that the Se-deficient diet leads to a decrease in motility ability and poor-quality semen as compared to increased Se in the diet [142]. Furthermore, one study showed that Se deficiency can lead to decreased production of spermatozoa. Generally, the total amount of Se in testes can be analyzed by observing the GPx4 concentration within them [143]. In another study, 69 infertile Scottish men received Se for 3 months combined with vitamins A, C, and E. A significant increase was found in sperm motility at the end of the clinical trial as compared to control patients [144]. As reported by Mintziori et al. [145], Se supplementation (<200 μg/day) is probably beneficial for men in terms of improving sperm motility. In other studies, it was found that supplementing the diet with organic Se at an amount of 0.6 and 0.9 ppm increases sperm motility and vitality in Thai-native chickens [146]. Moreover, another study suggested that the level of mitochondrial glutathione peroxidase (mGPx4) that becomes prominent during puberty neutralizes free radicals created throughout a succession of redox reactions along inside mitochondria. Furthermore, the increased level of mGPx4, manages the appearance and arrangement of the mitochondrial casing by creating a transverse connection through itself, as well as additional proteins [147]. When the mGPx4 concentration declinea, the structural consistency of sperms reduces, resulting in infertility. Additionally, research has revealed that a low amount of mGPx4 was present in infertile males [148].

## The Role of Se in Pregnancy

The requirement for several nutrients increases during the gestation period due to several metabolic changes in the body [149]. The reduced Se blood concentration during pregnancy is frequently associated with reduced activity of the GPx enzyme. Several authors have demonstrated that whole-blood and red blood cell enzyme activity decreases throughout pregnancy due to Se deficiency in the blood. According to previous studies, it has been proved that the concentrations of Se throughout the gestation period were found to be strongly reduced. GPx activity was found to be substantially reduced during the first trimester and this low level was sustained until the third trimester, with a slight drop during delivery of the fetus [150, 151]. Deficiencies of Se in pregnant women could harm the growth of fetus, especially it affects the nervous system of growing fetus. Moreover, case-control analysis of expectant mothers found a significant relationship between lower levels of Se and neural tube disabilities [8]. Oxidative stress is believed to arise due to Se deficiencies throughout pregnancy, leading to miscarriages, premature birth, cholestasis, intrauterine retardations, preeclampsia, thyroid disorders, and diabetes mellitus [152].

## Se Status in Middle Eastern Countries

Se status in Middle Eastern countries is widely variable, as Se is found less in some countries and more in others. This variability is apparent even among provinces in the same country. These variations can be related to varying concentrations of the Se in the soil where food is grown. In this section of the review paper, we summarize the status of Se and its intake among Middle Eastern countries based on the available literature. Here, it should be noted that the majority of studies that assessed the concentrations of Se in biological samples were case-controlled with small sample sizes, and cannot be generalized to the whole population. The experiments based on Se around the Middle East have various findings [153].

## Se Status in the Kingdom of Saudi Arabia

Studies in the Kingdom of Saudi Arabia (KSA) studies have shown that soil Se levels and food which is grown in KSA regions have low to poor Se concentrations [152]. According to the data presented by Al-Saleh [154], the content of Se in the soil in KSA (in Al-Kharj, which is located in close proximity to Riyadh city) ranges from 0.1 to 0.11 mg/kg. The KSA government is actively making efforts to ensure food safety in the Kingdom by promoting home-grown items. Even so, food imports are a major part of the local supply, almost all originating from the US, whose soil Se grades appear to be higher to adequate [153]. The main factors contributing to Se consumption in KSA are cereals and baked products, followed by fish and meat products, and dairy products [155, 156]. With the aim of helping to increase Se consumption, a study has been conducted by students of King Abdul-Aziz University study at Jeddah on the Se content of different food items [157, 158]. The major food groups tested in the experiment were cereal products, legumes, and meats. Moreover, for infants residing in KSA, the consumption of Se is achieved through infant formulas imported from Europe to ensure adequate intake of Se [159]. Conversely, many breast-fed infants do not get enough Se [154]. Food choices may vary among cities and suburbs and various areas of the state where there may be variations in the accessibility of some imported goods. This may have a significant effect on Se levels observed in the KSA community [152]. It is worth noting that the estimated Se consumption in Saudi Arabia in a study by Al-Ahmary [158] was 75–122 μg/person/day, and this level was close to the recommended level (72 μg/person/day). It is worth noting that in the studies conducted by Al-Othman et al. [156] the daily Se consumption by Saudis in the city of Riyadh was greater than for Sweden (44 μg/person/day) or Greece (39 μg/person/day). Nevertheless, it is important to carry out further research aimed at determining the bioavailability of Se in food in order to correctly determine the value of this element in the diet.

## Se Status in Jordan

There are currently no scientific data on the Se content of soil in Jordan. Only the content of Se in the water has been determined. A groundwater survey was carried out to assess Se levels in several aquifers at Amman Zarqa Basin, which is regareded as the most populated and industrialized basin in Jordan. Se levels ranged from 0.09 to 0.74 mg/L across different aquifers, with an average of 24 μg/L, which exceeds the WHO recommended drinking water threshold [160]. Another publication by Mistry et al. [161] states that the average content of Se in water is 0.308 mg/L. Thus, it can be noted that the content of this element in soil and food grown in these areas may be very low. A later case-control analysis in patients with colorectal cancer tested Se consumption (38.75 ±11.42 μg/day) which was significantly lower than the control (59.26 ±8.91 μg/day). It should be noted that the recommended daily allowances (RDA) for Se should be at the level of 60–70 μg/day [162]. Concentrations of Se have, however, been examined in two studies using samples of blood and hair [163]. Blood concentrations of about 187 μg/L were considered to be comparatively large in nonsmokers [164]. According to the literature, it has been revealed that in Jordan the assessment of seasonal trends in distributions of Se displayed greater levels during the dry season. The strategy including all basic of potential sources and factors leading to the water content of Se has been reviewed. Serious adverse risk analysis and exposure among residents using spring water was also estimated. The approximate daily average dose and noncarcinogenic hazard measurement values for Se exposure were below the level of concern for negative health effects [165]. In conclusion, it should be noted that the local grain harvest in Jordan is largely insufficient to meet demand. That is why Jordan is dependent on food imports. In addition, the presented data may not reflect the actual nutritional status of the population of this country with regard to this trace element (Se) [166]; therefore, it is worth carrying out further research in this direction to determine the level of this element in Jordan.

## Se Status in Turkey

The content of Se in soil in various regions of Turkey ranges from 0.01 to 0.08 mg/kg [167]. The consumption of bread in Turkey by the average person resident ranges from 150 to 450 g/day, which is almost half of the dry diet [168, 169]. Bearing in mind that wheat is the staple food in this country, it seems that the availability of dietary Se for humans will depend on the content of this element in this plant [169]. The average levels of Se in white, whole grain. and maize wheat bread were respectively 1.149, 1.204, and 2.023 μg/kg [168]. The Se concentration was measured in the colostrum and mature breast milk samples of the healthy lactating women in Turkey. It was found that the Se content was below the international reference range set at 18.5 μg/L among all the analyzed samples during the whole lactation period [152]. Furthermore, various samples from dairy milk products were also analyzed. The study found that goat milk had the highest Se content, and cow milk had the lowest content of Se [8]. All dairy products were analyzed in another study, where it was revealed that Se concentrations varied based on the type of food. Food samples containing butter and cheese had higher concentrations, whereas other products such as milk, ice cream, and yogurt had almost no Se [170]. Depending on the region, the daily Se intake generally ranges from 4 to 84 μg [169]. Different data provided by Aras et al. [171] indicate that the daily intake of Se is 20–53 μg/day. In the case of the estimated Se intake in children, this was reported as 30–40 μg/day [172]. Most studies from Turkey have shown that the community has an suboptimal Se level [173, 174]. However, other studies documented concentrations of plasma [70] and serum Se slightly above 120 μg/L [173].

## Se Status in Libya

Libya is located on the North African coast, bordered by the Mediterranean Sea to the north. In Libya, groundwater is the primary source of water for drinking supplies and for agriculture [175]. Elemental analysis of fields from two distinct agricultural land sectors in Libya was performed to calculate the number of certain trace elements utilizing their long-lived radioactive isotopes. This showed that the amount of Se in Libyan soil is much lower than in other countries. The average content of Se in the Earth’s crust is 0.4 μg/g. The findings of the experiment indicated that Se concentrations in Libyan clay surface soil (0.16–0.62 μg/g) were greater than in sandy soil (0.09–0.22 μg/g) [176]. The Se content of various Libyan food items was calculated, including a number of types of local and imported food, such as wheat, rice, bread, nuts, tea, coffee, and commonly used additives such as black and red pepper. The results suggested that the level of Se in imported products, such as rice, wheat, and wheat products, varies significantly with both the species of plants, as well as the condition of the lands in which they were grown. Moreover, the calculated and analyzed dietary Se levels tend to be lower throughout the Libyan community than is recommended. Dosing of Se in Libyan diets is strongly recommended through introducing Se to Libyan fields as a fertilizer application, especially for cereal schemes [177].

## Se Status in Junhuriyah Misr-Al-Arabiya (Egypt)

Se is not mentioned in the Egyptian food formulation table. Therefore, limited studies have been undertaken to evaluate Se in various food groups, to support a formal decision on Egyptians’ dietary Se intake [178]. Consumption of 8.3 mg/day among healthy children in Egypt has been reported [179]. Case-control studies were performed primarily in children living in Egypt and Se levels of 65–83 μg/L were documented [8]. It should be noted that skilful research and processes aimed at determining the enrichment of this element in the diet will likely increase Se supplementation by people living in Egypt. To sum up, the condition of proper human nutrition is complete coverage of the body’s energy requirements and individual nutrients needed for physical and mental development and maintaining full health. Appropriate balancing of Se in the diet can help to minimize the occurrence of various disease states.

## Se Status in Iran

The content of soil-Se in Iran ranges from 0.04 to 0.45 mg/kg [180]. Considering that rice is one of the most important foods in Iran, it may be of interest to determine the content of Se in this product. Rahimzadeh-Barzoki et al. [181] investigated the association between Se concentration in rice and the rate of esophageal malignancy in Golestan province, Iran. They measured the Se content of various samples of rice. The mean Se level in rice samples was 0.229 mg/kg. Moreover, Se levels in Iranian rice samples were significantly higher than in other countries [178] (0.020 mg/kg and 0.05 mg/kg in Italy and Korea, respectively) [182]. Extensive studies on Se content in different groups including adolescents, adults, and the elderly have been conducted in Iran [178, 180]. The intake in adults and children was found to be sufficient, whereas postmenopausal women had a significantly lower intake than the RDA, which is 55 μg/day [180]. Another study revealed that the serum selenium concentration ranged from 58 to 123 μg/L in infants, adults, and expectant mothers [183]. Mirzaeian et al. [184] assess Se intakes in female students in Isfahan, Iran. Food intake analysis indicated that the amount of Se was 54.5 μg/day, which is not significantly different from the recommended value (55 μg/day). According to other studies, it can be said that Se intake was found to be adequate in Iranian children and adults [8, 152, 185].

## Se Status in Qatar (Dawlat Qatar)

In Qatar, there have been no direct studies to determine Se intake among the Qatari population. The concentration of Se in soils is low (0.12–0.77 mg/kg) [186]. According to Qatar General Electricity & Water Company (Kahramaa), Se is not believed to be present in the water system in Qatar [187]. However, studies presented by Sharm [186] showed that the selenium content in groundwater is from 0.6 to 80 μg/L [186]. Besides, in Qatar’s primary staple food plant, a survey of Se in imported rice concluded that rice makes up more than 100% of the reference nutritive intake (RNI) of Se, which was 30 μg/day for Qatari citizens. However, for foreign residents of Qatar (more than 80% of the population) who ingested much less rice, the percentages varied with sex and rice variety, and all were below 100% of RNI Se. Further research included rice-based infant cereals in Qatar, which were found to provide approximately 63% of RNI Se based on the daily portion recommended [8]. This may mean that the use of rice in Qatar contributes greatly to daily intake of Se. Even so, Se status was not evaluated in any of the several available studies in Qatar.

## Conclusions

Se is an important trace mineral for successful functionality of human and animal species. Unlike many other trace elements, it is an element with a very limited quantitative range of concentrations between deficiency and physiological status, as well as toxic concentrations. Overall, the findings of this systematic analysis show that the intake and status of Se are inadequate in Middle Eastern countries with much less consistency as per current measures of sustainability. These findings, together with increasing awareness of the importance of Se to general health, require additional work, primarily on establishing an accepted range of blood Se concentration or other indicators to determine appropriate Se consumption and, thereby, to guarantee adequate Se supplementation in groups at risk of poor Se intake. More work should be done on the evaluation of Se content in the Middle Eastern population and its impacts on the health of individuals living in those countries.

## References

[1] Bhattacharya PT, Misra SR, Hussain M (2016) Nutritional aspects of essential trace elements in oral health and disease: an extensive review. Scientifica, Article ID 5464373. 10.1155/2016/5464373 10.1155/2016/5464373 PMC4940574 27433374

[2] Bano I, Sajjad H, Talpur MSH, Leghari A, Mirbahar KH (2016). Role of selenium on oxidative stress and male reproductive system. Pak J Biochem Mol Biol.

[3] Lenz M, Lens PN (2009). The essential toxin: the changing perception of selenium in environmental sciences. Sci Total Environ.

[4] Kieliszek M, Błażejak S (2013). Selenium: significance, and outlook for supplementation. Nutrition.

[5] Kieliszek M (2019). Selenium—fascinating microelement, properties and sources in food. Molecules.

[6] Kieliszek M, Błażejak S (2016). Current knowledge on the importance of selenium in food for living organisms: a review. Molecules.

[7] Combs F (2015). Biomarkers of selenium status. Nutrients.

[8] Ibrahim SA, Kerkadi A, Agouni A (2019). Selenium and health: an update on the situation in the Middle East and North Africa. Nutrients.

[9] Avery JC, Hoffmann PR (2018). Selenium, selenoproteins, and immunity. Nutrients.

[10] Tomasi N, Pinton R, Gottardi S, Mimmo T, Scampicchio M, Cesco S (2015). Selenium fortification of hydroponically grown corn salad (*Valerianella locusta*). Crop Pasture Sci.

[11] Stillwell RJ, Berry MJ (2005). Expanding the repertoire of the eukaryotic selenoproteome. PNAS.

[12] Dagnell M, Frijhoff J, Pader I, Augsten M, Boivin B, Xu J (2013). Selective activation of oxidized PTP1B by the thioredoxin system modulates PDGF-β receptor tyrosine kinase signaling. PNAS.

[13] Steinbrenner H, Sies H (2013). Selenium homeostasis and antioxidant selenoproteins in brain: implications for disorders in the central nervous system. Arch Biochem Biophys.

[14] Burk RF, Hill KE (2009). Selenoprotein P—expression, functions, and roles in mammals. Biochim Biophys Acta, Gen Subj.

[15] Brodin O, Hackler J, Misra S, Wendt S, Sun Q, Laaf E (2020). Selenoprotein P as biomarker of selenium status in clinical trials with therapeutic dosages of selenite. Nutrients.

[16] Fairweather-Tait SJ, Bao Y, Broadley MR, Collings R, Ford D, Hesketh JE, Hurst R (2011). Selenium in human health and disease. Antioxid Redox Signal.

[17] di Giuseppe R, Koch M, Nöthlings U, Kastenmüller G, Artati A, Adamski J, Gunnar J, Lieb W (2019). Metabolomics signature associated with circulating serum selenoprotein P levels. Endocrine.

[18] Traulsen H, Steinbrenner H, Buchczyk DP, Klotz LO, Sies H (2004). Selenoprotein P protects low-density lipoprotein against oxidation. Free Radic Res.

[19] Talbi W, Ghazouani T, Braconi D, Ben Abdallah R, Raboudi F, Santucci A, Fattouch S (2019). Effects of selenium on oxidative damage and antioxidant enzymes of eukaryotic cells: wine *Saccharomyces cerevisiae*. J Appl Microbiol.

[20] Burk RF, Hill KE, Read R, Bellew T (1991). Response of rat selenoprotein P to selenium administration and fate of its selenium. Am J Physiol Endocrinol Metab.

[21] Hill KE, Zhou J, McMahan WJ, Motley AK, Atkins JF, Gesteland RF, Burk RF (2003). Deletion of selenoprotein P alters distribution of selenium in the mouse. J Biol Chem.

[22] Burk RF, Hill KE, Motley AK, Austin LM, Norsworthy BK (2006). Deletion of selenoprotein P upregulates urinary selenium excretion and depresses whole-body selenium content. Biochim Biophys Acta, Gen Subj.

[23] Grundner-Culemann E, Martin GW, Harney JW, Berry MJ (1999). Two distinct SECIS structures capable of directing selenocysteine incorporation in eukaryotes. RNA.

[24] Yeh JY, Vendeland SC, Gu QP, Butler JA, Ou BR, Whanger PD (1997). Dietary selenium increases selenoprotein W levels in rat tissues. J Nutr.

[25] Yildirim S, Ozkan C, Huyut Z, Çınar A (2019). Detection of Se, vit. E, vit. A, MDA, 8-OHdG, and CoQ10 levels and histopathological changes in heart tissue in sheep with white muscle disease. Biol Trace Elem Res.

[26] Chen YL, Yang KC, Chang HH, Lee LT, Lu CW, Huang KC (2014). Low serum selenium level is associated with low muscle mass in the community-dwelling elderly. J Am Med Dir Assoc.

[27] Howard MT, Carlson BA, Anderson CB, Hatfield DL (2013). Translational redefinition of UGA codons is regulated by selenium availability. J Biol Chem.

[28] Beilstein MA, Vendeland SC, Barofsky E, Jensen ON, Whanger PD (1996). Selenoprotein W of rat muscle binds glutathione and an unknown small molecular weight moiety. J Inorg Biochem.

[29] Madeja Z, Sroka J, Nyström C, Björkhem-Bergman L, Nordman T, Damdimopoulos A (2005). The role of thioredoxin reductase activity in selenium-induced cytotoxicity. Biochem Pharmacol.

[30] Weekley CM, Harris HH (2013). Which form is that? The importance of selenium speciation and metabolism in the prevention and treatment of disease. Chem Soc Rev.

[31] Merrill GF, Dowell P, Pearson GD (1999). The human p53 negative regulatory domain mediates inhibition of reporter gene transactivation in yeast lacking thioredoxin reductase. Cancer Res.

[32] Hatfield DL, Yoo MH, Carlson BA, Gladyshev VN (2009). Selenoproteins that function in cancer prevention and promotion. Biochim Biophys Acta, Gen Subj.

[33] Fath MA, Ahmad IM, Smith CJ, Spence J, Spitz DR (2011). Enhancement of carboplatin-mediated lung cancer cell killing by simultaneous disruption of glutathione and thioredoxin metabolism. Clin Cancer Res.

[34] Kalantari P, Narayan V, Natarajan SK, Muralidhar K, Gandhi UH, Vunta H (2008). Thioredoxin reductase-1 negatively regulates HIV-1 transactivating protein Tat-dependent transcription in human macrophages. J Biol Chem.

[35] Toppo S, Vanin S, Bosello V, Tosatto SCE (2008). Evolutionary and structural insights into the multifaceted glutathione peroxidase (Gpx) superfamily. Antioxid Redox Signal.

[36] Kieliszek M, Błażejak S, Bzducha-Wróbel A, Kot A (2019). Effect of selenium on growth and antioxidative system of yeast cells. Mol Biol Rep.

[37] Jiao Y, Wang Y, Guo S, Wang G (2017). Glutathione peroxidases as oncotargets. Oncotarget.

[38] Liu C, Yan Q, Gao C, Lin L, Wei J (2021). Study on antioxidant effect of recombinant glutathione peroxidase 1. Int J Biol Macromol.

[39] Alhasan R, Kharma A, Leroy P, Jacob C, Gaucher C (2019). Selenium donors at the junction of inflammatory diseases. Curr Pharm Des.

[40] Köhrle J, Brigelius-Flohé R, Böck A, Gärtner R, Meyer O, Flohé L (2000). Selenium in biology: facts and medical perspectives. Biol Chem.

[41] Maiorino M, Scapin M, Ursini F, Biasolo M, Bosello V, Flohé L (2003). Distinct promoters determine alternative transcription of gpx-4 into phospholipid-hydroperoxide glutathione peroxidase variants. J Biol Chem.

[42] Brigelius-Flohé R, Maiorino M (2003). Glutathione peroxidases. Biochim Biophys Acta, Gen Subj.

[43] Reich HJ, Hondal RJ (2016). Why nature chose selenium. ACS Chem Biol.

[44] Tan LC, Nancharaiah YV, van Hullebusch ED, Lens PN (2016). Selenium: environmental significance, pollution, and biological treatment technologies. Biotechnol Adv.

[45] Wen H, Carignan J (2007). Reviews on atmospheric selenium: emissions, speciation and fate. Atmos Environ.

[46] Rodriguez M, Rivero V, Ballesta RJ (2005). Selenium distribution in topsoils and plants of a semiarid Mediterranean environment. Environ Geochem Health.

[47] Zhou X, Li Y, Lai F (2018). Effects of different water management on absorption and accumulation of selenium in rice. Saudi J Biol Sci.

[48] Niedzielski, Siepak M, Siepak J (2000). Występowanie i zawartość arsenu, antymonu i selenu w wodach i innych elementach środowiska. Rocznik Ochrona Środowiska.

[49] He Y, Xiang Y, Zhou Y, Yang Y, Zhang J, Huang H (2018). Selenium contamination, consequences and remediation techniques in water and soils: a review. Environ Res.

[50] Kieliszek M, Błażejak S, Piwowarek K, Brzezicka K (2018). Equilibrium modeling of selenium binding from aqueous solutions by *Candida utilis* ATCC 9950 yeasts. 3 Biotech.

[51] Etim EU (2017). Occurrence and distribution of arsenic, antimony and selenium in shallow groundwater systems of Ibadan metropolis, southwestern Nigerian. J Heal Pollut.

[52] Stefaniak J, Dutta A, Verbinnen B, Shakya M, Rene ER (2018). Selenium removal from mining and process wastewater: a systematic review of available technologies. J Water Supply Res T.

[53] Malhotra M, Pal M, Pal P (2020). A response surface optimized nanofiltration-based system for efficient removal of selenium from drinking Water. J Water Process Eng.

[54] Mehdi Y, Hornick JL, Istasse L, Dufrasne I (2013). Selenium in the environment, metabolism and involvement in body functions. Molecules.

[55] Reynolds RJB, Cappa JJ, Pilon-Smits EAH, Pilon-Smits E, Winkel L, Lin ZQ (2017). Evolutionary aspects of plant selenium accumulation. Selenium in plants. Plant ecophysiology.

[56] Giger-Reverdin S (1995). Review of the main methods of cell wall estimation: interest and limits for ruminants. Anim Feed Sci Technol.

[57] Pilon-Smits EA (2019). On the ecology of selenium accumulation in plants. Plants.

[58] Hu T, Li H, Li J, Zhao G, Wu W, Liu L (2018). Absorption and bio-transformation of selenium nanoparticles by wheat seedlings (*Triticum aestivum* L.). Front Plant Sci.

[59] Navarro-Alarcon M, Cabrera-Vique C (2008). Selenium in food and the human body: a review. Sci Total Environ.

[60] Kieliszek M, Błażejak S (2018). Speciation analysis of selenium in *Candida utilis* yeast cells using HPLC-ICP-MS and UHPLC-ESI-Orbitrap MS techniques. Appl Sci.

[61] Casey CE, Guthrie BE, Friend GM, Robinson MF (2013) Archives of environmental health : an international journal selenium in human tissues from New Zealand Selenium in human tissues from New Zealand 1-4. 10.1080/00039896.1982.10667551. 10.1080/00039896.1982.10667551 7092329

[62] Navarro-Alarcon M, López-Martınez MC (2000). Essentiality of selenium in the human body: relationship with different diseases. Sci Total Environ.

[63] Hasani M, Djalalinia S, Khazdooz M, Asayesh H, Zarei M, Gorabi AM (2019). Effect of selenium supplementation on antioxidant markers: a systematic review and meta-analysis of randomized controlled trials. Hormones.

[64] Holben DH, Smith AM, Ilich JZ, Landoll JD, Holcomb JP, Matkovic V (2002). Selenium intakes, absorption, retention, and status in adolescent girls. J Am Diet Assoc.

[65] Finley JW (2006). Brief critical review bioavailability of selenium from foods. Nutr Rev.

[66] Kipp AP, Strohm D, Brigelius-Flohé R, Schomburg L, Bechthold A, Leschik-Bonnet E, Heseker H (2015). Revised reference values for selenium intake. J Trace Elem Med Biol.

[67] Institute of Medicine (US) 2000 Panel on Dietary Antioxidants and Related Compounds. Dietary Reference Intakes for Vitamin C, Vitamin E, Selenium, and Carotenoids. Washington (DC): National Academies Press (US); 7, Selenium. Available from: https://www.ncbi.nlm.nih.gov/books/NBK225470/. Accessed 01 Dec 2021

[68] Fisinin VI, Papazyan TT, Surai PF (2009). Producing selenium-enriched eggs and meat to improve the selenium status of the general population. Crit Rev Biotechnol.

[69] Bourre JM, Galea F (2006). An important source of omega-3 fatty acids, vitamins D and E, carotenoids, iodine and selenium: a new natural multi-enriched egg. J Nutr Health Aging.

[70] Steinbrenner H, Al-Quraishy S, Dkhil M, Wunderlich F, Sies H (2015). Dietary selenium in adjuvant therapy of viral. Adv Nutr.

[71] Tinggi U (2013). Essentiality and toxicity of selenium and its status in Australia: a review. Toxicol Lett.

[72] Fairweather-Tait SJ, Collings R, Hurst R (2010). Selenium bioavailability: current knowledge and future research requirements. Am J Clin Nutr.

[73] Alves MR, Starling AL, Kanufre VC, Soares RD, Norton RDC, Aguiar MJ, Januario JN (2012). Selenium intake and nutritional status of children with phenylketonuria in Minas Gerais. Brazil J Pediatr (Rio J).

[74] Eroglu C, Unal D, Cetin A, Orhan O, Sivgin S, Oztürk A (2012). Effect of serum selenium levels on radiotherapy-related toxicity in patients undergoing radiotherapy for head and neck cancer. Anticancer Res.

[75] Yao Y, Pei F, Kang P (2011). Selenium, iodine, and the relation with Kashin–Beck disease. Nutrition.

[76] Hadrup N, Ravn-Haren G (2020). Acute human toxicity and mortality after selenium ingestion: a review. J Trace Elem Med Biol.

[77] Rayman MP (2008). Food-chain selenium and human health: emphasis on intake. Br J Nutr.

[78] Thiry C, Ruttens A, De Temmerman L, Schneider YJ, Pussemier L (2012). Current knowledge in species-related bioavailability of selenium in food. Food Chem.

[79] Dhanya BL, Swathy RP, Indira M (2014). Selenium downregulates oxidative stress-induced activation of leukotriene pathway in experimental rats with diabetic cardiac hypertrophy. Biol Trace Elem Res.

[80] Tong C, Li P, Yu LH, Li L, Li K, Chen Y (2020). Selenium-rich yeast attenuates ochratoxin A-induced small intestinal injury in broiler chickens by activating the Nrf2 pathway and inhibiting NF-KB activation. J Funct Foods.

[81] Schomburg L, Schweizer U (2009). Biochimica et Biophysica Acta Hierarchical regulation of selenoprotein expression and sex-specific effects of selenium. Biochim Biophys Acta, Gen Subj.

[82] Michalke B (2004). Selenium speciation in human serum of cystic fibrosis patients compared to serum from healthy persons. J Chromatogr A.

[83] Ai P, Lei S, Zhou F, Chen S, Zhang Y (2020). Selenium levels and skin diseases: systematic review and meta-analysis. J Trace Elem Med Biol.

[84] Gao Y, Pagnon J, Feng HC, Konstantopolous N, Jowett JB, Walder K, Collier GR (2007). Secretion of the glucose-regulated selenoprotein SEPS1 from hepatoma cells. Biochem Biophys Res Commun.

[85] Shea-Donohue T, Qin B, Smith A (2017). Parasites, nutrition, immune responses and biology of metabolic tissues. Parasite Immunol.

[86] Koeberle SC, Kipp AP, Michalke B (2018). Selenium and inflammatory mediators. Selenium. Molecular and integrative toxicology.

[87] Gao Y, Hannan NR, Wanyonyi S, Konstantopolous N, Pagnon J, Feng HC (2006). Activation of the selenoprotein SEPS1 gene expression by pro-inflammatory cytokines in HepG2 cells. Cytokine.

[88] Bellinger FP, Raman AV, Reeves MA, Berry MJ (2009). Regulation and function of selenoproteins in human disease. Biochem J.

[89] Gladyshev VN, Factor VM, Housseau F, Hatfield DL (1998). Contrasting patterns of regulation of the antioxidant selenoproteins, thioredoxin reductase, and glutathione peroxidase, in cancer cells. Biochem Biophys Res Commun.

[90] Micke O, Schomburg L, Buentzel J, Kisters K, Muecke R (2009). Selenium in oncology: from chemistry to clinics. Molecules.

[91] Hawkes WC, Kelley DS, Taylor PC (2001). The effects of dietary selenium on the immune system in healthy men. Biol Trace Elem Res.

[92] Gladyshev VN, Jeang KT, Stadtman TC (1996). Selenocysteine, identified as the penultimate C-terminal residue in human T-cell thioredoxin reductase, corresponds to TGA in the human placental gene. PNAS.

[93] Taylor EW (1995). Selenium and cellular immunity. Evidence thatselenoproteins may be encoded in the +1 reading frameoverlapping the human CD4, CD8, and HLA-DR genes. Biol Trace Elem Res.

[94] Nomura AM, Lee J, Stemmermann GN, Combs GF (2000). Serum selenium and subsequent risk of prostate cancer. Cancer Epidemiol Biomark Prev.

[95] Jiang C, Wang Z, Ganther H, Lu J (2001). Caspases as key executors of methyl selenium-induced apoptosis (anoikis) of DU-145 prostate cancer cells. Cancer Res.

[96] Yoon SO, Kim MM, Chung AS (2001). Inhibitory effect of selenite on invasion of HT1080 tumor cells. J Biol Chem.

[97] Ichimura Y, Habuchi T, Tsuchiya N, Wang L, Oyama C (2004). Increased risk of bladder cancer associated with a glutathione peroxidase 1 codon 198 variant. J Urol.

[98] Combs GF, Gray WP (1998). Chemopreventive agents: selenium. Pharmacol Ther.

[99] Kuria A, Fang X, Li M, Han H, He J, Aaseth JO, Cao Y (2020). Does dietary intake of selenium protect against cancer? A systematic review and meta-analysis of population-based prospective studies. Crit Rev Food Sci Nutr.

[100] Hashemi SM, Mashhadi M, Moghaddam AA, Yousefi J, Mofrad AD, Sadeghi M, Allahyari A (2017). The relationship between serum selenium and zinc with gastroesophageal cancers in the Southeast of Iran. Indian J Med Paediatr Oncol.

[101] Wallenberg M, Misra S, Björnstedt M (2014). Selenium cytotoxicity in cancer. Basic Clin Pharmacol Toxicol.

[102] Chen YC, Sandeep Prabhu K, Mastro AM (2013). Is selenium a potential treatment for cancer metastasis?. Nutrients.

[103] Benstoem C, Goetzenich A, Kraemer S, Borosch S, Manzanares W, Hardy G, Stoppe C (2015). Selenium and its supplementation in cardiovascular disease—what do we know?. Nutrients.

[104] Thomson CD (2004). Assessment of requirements for selenium and adequacy of selenium status: a review. Eur J Clin Nutr.

[105] Kuria A, Tian H, Li M, Wang Y, Aaseth JO, Zang J, Cao Y (2020) Selenium status in the body and cardiovascular disease: a systematic review and meta-analysis. Crit Rev Food Sci Nutr:1–10. 10.1080/10408398.2020.1803200 10.1080/10408398.2020.1803200 32799545

[106] Jenkins DJ, Kitts D, Giovannucci EL, Sahye-Pudaruth S, Paquette M, Blanco Mejia S (2020). Selenium, antioxidants, cardiovascular disease, and all-cause mortality: a systematic review and meta-analysis of randomized controlled trials. Am J Clin Nutr.

[107] Gharipour M, Sadeghi M, Behmanesh M, Salehi M, Nezafati P, Gharipour A (2017). Selenium homeostasis and clustering of cardiovascular risk factors: a systematic review. Acta Biomed.

[108] Stranges S, Marshall JR, Natarajan R, Donahue RP, Trevisan M, Combs GF (2007). Annals of internal medicine article effects of long-term selenium supplementation on the incidence of type 2 diabetes. Ann Intern Med.

[109] Maulik N, Das DK (2008). Emerging potential of thioredoxin and thioredoxin interacting proteins in various disease conditions. Biochim Biophys Acta, Gen Subj.

[110] Ago T, Sadoshima J (2006). Thioredoxin and ventricular remodeling. J Mol Cell Cardiol.

[111] Dhingra S, Bansal MP (2006). Attenuation of LDL receptor gene expression by selenium deficiency during hypercholesterolemia. Mol Cell Biochem.

[112] Loef M, Schrauzer GN, Walach H (2011). Selenium and alzheimer’s disease: a systematic review. J Alzheimers Dis.

[113] Castaño A, Ayala A, Rodríguez-Gómez JA, Herrera AJ, Cano J, Machado A (1997). Low selenium diet increases the dopamine turnover in prefrontal cortex of the rat. Neurochem Int.

[114] Gao S, Jin Y, Hall KS, Liang C, Unverzagt FW, Ji R (2007). Selenium level and cognitive function in rural elderly Chinese. Am J Epidemiol.

[115] Wenstrup D, Ehman WD, Markesbery WR (1990). Trace element imbalances in isolated subcellular fractions of Alzheimer’s disease brains. Brain Res.

[116] Reddy VS, Bukke S, Dutt N, Rana P, Pandey AK (2017). A systematic review and meta-analysis of the circulatory, erythrocellular and CSF selenium levels in Alzheimer's disease: a metal meta-analysis (AMMA study-I). J Trace Elem Med Biol.

[117] da Silva Leme AGH, Cardoso BR (2020) Selenium and Alzheimer's disease. In: Genetics, neurology, behavior, and diet in dementia. Academic Press, pp 739–748. 10.1016/B978-0-12-815868-5.00047-5

[118] Tamtaji OR, Heidari-Soureshjani R, Mirhosseini N, Kouchaki E, Bahmani F, Aghadavod E (2019). Probiotic and selenium co-supplementation, and the effects on clinical, metabolic and genetic status in Alzheimer's disease: a randomized, double-blind, controlled trial. Clin Nutr.

[119] Reeves MA, Bellinger FP, Berry MJ (2010). The neuroprotective functions of selenoprotein M and its role in cytosolic calcium regulation. Antioxid Redox Signal.

[120] LaFerla FM (2002). Calcium dyshomeostasis and intracellular signalling in alzheimer’s disease. Nat Rev Neurosci.

[121] Wang Y, Shi Y, Wei H (2017). Calcium dysregulation in Alzheimer’s disease: a target for new drug development. J Alzheimers Dis.

[122] Kim J, Chung HS, Choi MK, Roh YK, Yoo HJ, Park JH (2019). Association between serum selenium level and the presence of diabetes mellitus: a meta-analysis of observational studies. Diabetes Metab J.

[123] Fontenelle LC, Feitosa MM, Morais JBS, Severo JS, Freitas TECD, Beserra JB et al (2018) The role of selenium in insulin resistance. Braz J Pharm Sci 54(1). 10.1590/s2175-97902018000100139

[124] Wang Y, Rijntjes E, Wu Q, Lv H, Gao C, Shi B, Schomburg L (2020). Selenium deficiency is linearly associated with hypoglycemia in healthy adults. Redox Biol.

[125] Bleys J, Navas-Acien A, Guallar E (2007). Serum selenium and diabetes in U.S. adults. Diabetes Care.

[126] Alizadeh M, Safaeiyan A, Ostadrahimi A, Estakhri R, Daneghian S, Ghaffari A, Gargari BP (2012). Effect of l-arginine and selenium added to a hypocaloric diet enriched with legumes on cardiovascular disease risk factors in women with central obesity: a randomized, double-blind, placebo-controlled trial. Ann Nutr Metab.

[127] Hofstee P, McKeating DR, Bartho LA, Anderson ST, Perkins AV, Cuffe JS (2020). Maternal selenium deficiency in mice alters offspring glucose metabolism and thyroid status in a sexually dimorphic manner. Nutrients.

[128] Liu Y, Zhao H, Zhang Q, Tang J, Li K, Xia XJ (2012). Prolonged dietary selenium deficiency or excess does not globally affect selenoprotein gene expression and/or protein production in various tissues of pigs. J Nutr.

[129] Kohler LN, Foote J, Kelley CP, Florea A, Shelly C, Chow HH (2018). Selenium and type 2 diabetes: systematic review. Nutrients.

[130] Derumeaux H, Valeix P, Castetbon K, Bensimon M, Boutron-Ruault MC, Arnaud J, Hercberg S (2003). Association of selenium with thyroid volume and echostructure in 35- to 60-year-old French adults. Eur J Endocrinol.

[131] Contempre B, Je D, Bebe NGO, CH T, AT D, Vanderpas J (1991). Effect of selenium supplementation in hypothyroid subjects of an iodine and selenium deficient area : the possible danger of indiscriminate supplementation of iodine-deficient subjects with selenium. J Clin Endocrinol Metab.

[132] Khong JJ, Goldstein RF, Sanders KM, Schneider H, Pope J, Burdon KP (2014). Serum selenium status in Graves’ disease with and without orbitopathy: a case-control study. Clin Endocrinol.

[133] Dehina N, Hofmann PJ, Behrends T, Eckstein A, Schomburg L (2016). Lack of association between selenium status and disease severity and activity in patients with Graves’ Ophthalmopathy. Eur Thyroid J.

[134] Wang Y, Zhao F, Rijntjes E, Wu L, Wu Q, Sui J (2018). Role of selenium intake for risk and development of hyperthyroidism. J Clin Endocrinol Metab.

[135] Marinò M, Menconi F, Dottore GR, Leo M, Marcocci C (2018). Selenium in Graves hyperthyroidism and orbitopathy. Ophthal Plast Reconstr Surg.

[136] Winther KH, Rayman MP, Bonnema SJ, Hegedüs L (2020). Selenium in thyroid disorders—essential knowledge for clinicians. Nat Rev Endocrinol.

[137] Sattar T (2021). Selenium role in reproduction, pregnant/postpartum women and neonates: a current study. Curr Nutr Food Sci.

[138] Alabi NS, Beilstein MA, Whanger PD (2000). Chemical forms of selenium present in rat and ram spermatozoa. Biol Trace Elem Res.

[139] Piagentini M, Silva DC, Dell'Aqua CPF, Moya-Araujo CF, Codognoto VM, Ramos AA, Oba E (2017). Effect of selenium supplementation on semen characteristics of Brazil's ram. Reprod Domest Anim.

[140] Stoedter M, Renko K, Hög A, Schomburg L (2010). Selenium controls the sex-specific immune response and selenoprotein expression during the acute-phase response in mice. Biochem J.

[141] Hawkes WC, Alkan Z, Wong K (2009). Selenium supplementation does not affect testicular selenium status or semen quality in North American men. J Androl.

[142] Ahsan U, Kamran Z, Raza I, Ahmad S, Babar W, Riaz MH, Iqbal Z (2014). Role of selenium in male reproduction—a review. Anim Reprod Sci.

[143] Foresta C, Flohé L, Garolla A, Roveri A, Ursini F, Maiorino M (2002). Male fertility is linked to the selenoprotein phospholipid hydroperoxide glutathione peroxidase. Biol Reprod.

[144] Scott R, Macpherson A, Yatest RWS, Hussain B, Dixon J (1998). The effect of oral selenium supplementation on human sperm motility. Br J Urol.

[145] Mintziori G, Mousiolis A, Duntas LH (2020). Evidence for a manifold role of selenium in infertility. Hormones.

[146] Chauychu-Noo N, Thananurak P, Boonkum W, Vongpralub T, Chankitisakul V (2021). Effect of organic selenium dietary supplementation on quality and fertility of cryopreserved chicken sperm. Cryobiol..

[147] Toppo S, Flohé L, Ursini F, Vanin S, Maiorino M (2009). Catalytic mechanisms and specificities of glutathione peroxidases: variations of a basic scheme. Biochim Biophys Acta, Gen Subj.

[148] Dosek A, Ohno H, Acs Z, Taylor AW, Radak Z (2007). High altitude and oxidative stress. Respir Physiol Neurobiol.

[149] Pieczyńska J, Grajeta H (2015). The role of selenium in human conception and pregnancy. J Trace Elem Med Biol.

[150] Mihailović M, Cvetković M, Ljubić A, Kosanović M, Nedeljković S, Jovanović I, Pešut O (2000). Selenium and malondialdehyde content and glutathione peroxidase activity in maternal and umbilical cord blood and amniotic fluid. Biol Trace Elem Res.

[151] Bedwal RS, Bahuguna A (1994). Zinc, copper and selenium in reproduction. Experientia.

[152] Stoffaneller R, Morse NL (2015). A review of dietary selenium intake and selenium status in Europe and the Middle East. Nutrients.

[153] Rayman MP (2012). Selenium and human health. Lancet.

[154] Al-Saleh I (2000). Trace elements selenium status in Saudi Arabia. J Trace Elem Med Biol.

[155] González S, Huerta JM, Fernández S, Patterson DM, Lasheras C (2006). Food intake and serum selenium concentration in elderly people. Ann Nutr Metab.

[156] Al-Othman AM, Al-Othman ZA, El-Desoky GE (2012). Daily intake of selenium and concentrations in blood of residents of Riyadh City, Saudi Arabia. Environ Geochem Health.

[157] Elsom R, Sanderson P, Hesketh JE, Jackson MJ, Fairweather-Tait SJ, Åkesson B (2006). Functional markers of selenium status: UK Food Standards Agency workshop report. Br J Nutr.

[158] Al-Ahmary KM (2009). Selenium content in selected foods from the Saudi Arabia market and estimation of the daily intake. Arab J Chem.

[159] Al-Saleh I, Al-Doush I (1997). Selenium levels in infant milk formula. Biometals.

[160] Kuisi MA, Abdel-Fattah A (2010). Groundwater vulnerability to selenium in semi-arid environments: Amman Zarqa Basin. Jordan Environ Geochem Health.

[161] Mistry HD, Kurlak LO, Young SD, Briley AL, Broughton Pipkin F, Baker PN, Poston L (2014). Maternal selenium, copper and zinc concentrations in pregnancy associated with small-for-gestational-age infants. Matern Child Nutr.

[162] Arafa MA, Waly MI, Jriesat S, Khafajei AA, Sallam S (2011). Dietary and lifestyle characteristics of colorectal cancer in Jordan: a case-control study. Asian Pac J Cancer Prev.

[163] Alqhazo M, Rashaid AB (2018). The concentrations of bioelements in the hair samples of Jordanian children who stutter. Int J Pediatr Otorhinolaryngol.

[164] Massadeh A, Gharibeh A, Omari K, Al-Momani I, Alomari A, Tumah H, Hayajneh W (2010). Simultaneous determination of Cd, Pb, Cu, Zn, and Se in human blood of Jordanian smokers by ICP-OES. Biol Trace Elem Res.

[165] Al-Taani AA, Batayneh A, El-Radaideh N, Al-Momani I, Rawabdeh A (2012). Monitoring of selenium concentrations in major springs of Yarmouk Basin, North Jordan. World Appl Sci J.

[166] Hammouh F, Zein S, Amr R, Ghazzawi H, Muharib D, Al Saad D, Subih H (2020) Assessment of dietary selenium intake of Jordanian adults in Madaba: a cross sectional study. Nutr Food Sci. 10.1108/NFS-11-2019-0337

[167] Beytut E, Karatas F, Beytut E (2002). Lambs with white muscle disease and selenium content of soil and meadow hay in the region of Kars, Turkey. Vet J.

[168] Gülfen M (2012). Selenium levels in breads from Sakarya, Turkey. Food Addit Contam Part B.

[169] Giray B, Hincal F (2002). Oxidative DNA base damage, antioxidant enzyme activities and selenium status in highly iodine-deficient goitrous children. Free Radic Res.

[170] Ayar A, Sert D, Akin N (2009). The trace metal levels in milk and dairy products consumed in middle Anatolia-Turkey. Environ Monit Assess.

[171] Aras NK, Nazli A, Zhang W, Chatt A (2001). Dietary intake of zinc and selenium in Turkey. J Radioanal Nucl Chem.

[172] Hincal F (2007). Trace elements in growth: iodine and selenium status of Turkish children. J Trace Elem Med Biol.

[173] Özdemir HS, Karadas F, Pappas AC, Cassey P, Oto G, Tuncer O (2008). The selenium levels of mothers and their neonates using hair, breast milk, meconium, and maternal and umbilical cord blood in Van Basin. Biol Trace Elem Res.

[174] Duffield AJ, Thomson CD, Hill KE, Williams S (1999). An estimation of selenium requirements for New Zealanders. Am J Clin Nutr.

[175] Abd El-Aziz SH (2017). Evaluation of groundwater quality for drinking and irrigation purposes in the north-western area of Libya (Aligeelat). Environ Earth Sci.

[176] El-Ghawi UM, Al-Fakhri SM, Al-Sadeq AA, Bejey MM, Doubali KK (2007). The level of selenium and some other trace elements in different Libyan arable soils using instrumental neutron activation analysis. Biol Trace Elem Res.

[177] El-Ghawi UM, Al-Sadeq AA, Bejey MM, Alamin MB (2005). Determination of selenium in Libyan food items using pseudocyclic instrumental neutron activation analysis. Biol Trace Elem Res.

[178] Moatkhef F, Ismail H, Agamy N (2020). Quantitative determination of selenium in the most common food items sold in Egypt. J Egypt Public Health Assoc.

[179] Meguid NA, Anwar M, Bjørklund G, Hashish A, Chirumbolo S, Hemimi M, Sultan E (2017). Dietary adequacy of Egyptian children with autism spectrum disorder compared to healthy developing children. Metab Brain Dis.

[180] Nazemi L, Nazmara S, Eshraghyan MR (2012). Selenium status in soil, water and essential crops of Iran. J Environ Health Sci Eng.

[181] Rahimzadeh-Barzoki H, Joshaghani H, Beirami S, Mansurian M, Semnani S, Roshandel G (2014). Selenium levels in rice samples from high and low risk areas for esophageal cancer. Saudi Med J.

[182] Atarodi B, Fotovat A (2015) Selenium status in Iran: a soil and human health point of view. In: Global advances in selenium research from theory to application: proceedings of the 4th international conference on selenium in the environment and human health 2015. CRC Press, p 91

[183] Safaralizadeh R, Kardar G, Pourpak Z (2005). Serum concentration of selenium in healthy individuals living in Tehran. Nutr J.

[184] Mirzaeian S, Ghiasvand R, Sadeghian F, Sheikhi M, Khosravi ZS, Askari G, Shiranian A, Yadegarfar G (2013). Assessing the micronutrient and macronutrient intakes in female students and comparing them with the set standard values. J Educ Health Promot.

[185] Safaralizadeh R, Sirjani M, Pourpak Z, Kardar G, Teimourian S, Shams S, Namdar Z, Kazemnejad A, Mostafa Moin Moin M (2007). Serum selenium concentration in healthy children living in Tehran. Biofactors.

[186] Shomar B (2015). Geochemistry of soil and groundwater in arid regions: Qatar as a case study. Groundw Sustain Dev.

[187] https://www.km.com.qa/MediaCenter/Publications/KAHRAMAA/Drinking/Water/Quality/Requirment.pdf (accessed on 10 December 2020)

